# CHRONIC PAIN, FEAR OF FALLING, AND RESTRICTED ACTIVITY DAYS IN AN OLDER POPULATION

**DOI:** 10.1093/geroni/igz038.066

**Published:** 2019-11-08

**Authors:** Suzanne Leveille

**Affiliations:** University of Massachusetts Boston, Boston, Massachusetts, United States

## Abstract

Both chronic pain and fear of falling can lead to activity restriction and increased fall risk among vulnerable elders. Little is known about pain characteristics that may be associated with fear of falling, contributing to restricted activity. We studied 765 adults aged ≥65y (mean=78.9y) in the MOBILIZE Boston Study, to evaluate the cross-sectional relationship between pain characteristics and fear of falling measured using the Falls Efficacy Scale (FES). In addition, we examined the impact of pain and fear of falling on restricted activity. We measured 3 domains of global pain: pain distribution (none, single site or multisite pain), and Brief Pain Inventory subscales of pain severity and pain interference. Restricted activity days (RADs) refer to the count of self-reported days of reduced activity due to illness or injury in the previous 12 months. We performed multivariable logistic regressions predicting fear of falling (FES<90/100) adjusted for sociodemographics, fall history and fall risk factors. Participants with multisite pain or moderate-to-high pain interference ratings were more likely to have fear of falling (adj.OR 1.97, 95%CI 1.05-3.67; adj.OR 4.02, 95%CI 2.0-8.06, respectively). Pain severity was not associated with FES. Older adults with multisite pain and fear of falling reported significantly more RADs than those with multisite pain without fear of falling (79±135 and 26±74 RADs, respectively; test for pain x FES interaction, p=0.01). Older adults with chronic pain have greater fear of falling which may contribute to restricted activity. Efforts are needed to increase activity and falls efficacy among older adults with chronic pain.

